# Supplementary figures and images for: Different Transcriptome Features of Peripheral Blood Mononuclear Cells in Non-Emphysematous Chronic Obstructive Pulmonary Disease
Source: Int J Mol Sci. 2023 Dec 20;25(1):66. doi: 10.3390/ijms25010066 (PMC10779039; doi:10.3390/ijms25010066)

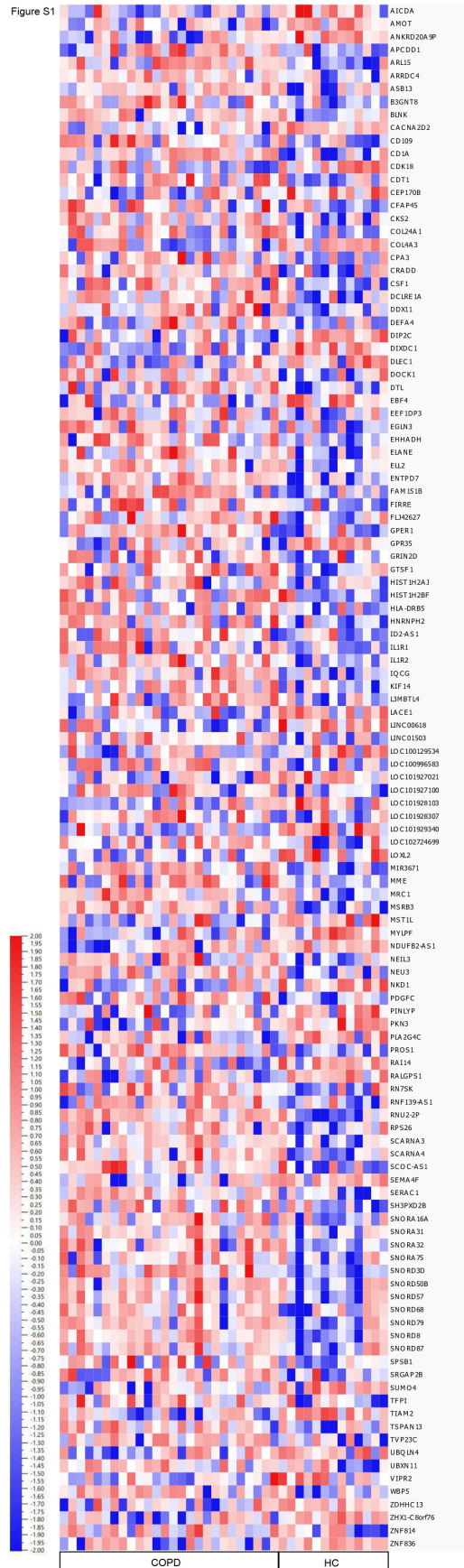

**Figure S1:** The details of Figure 3 are shown

Figure S2

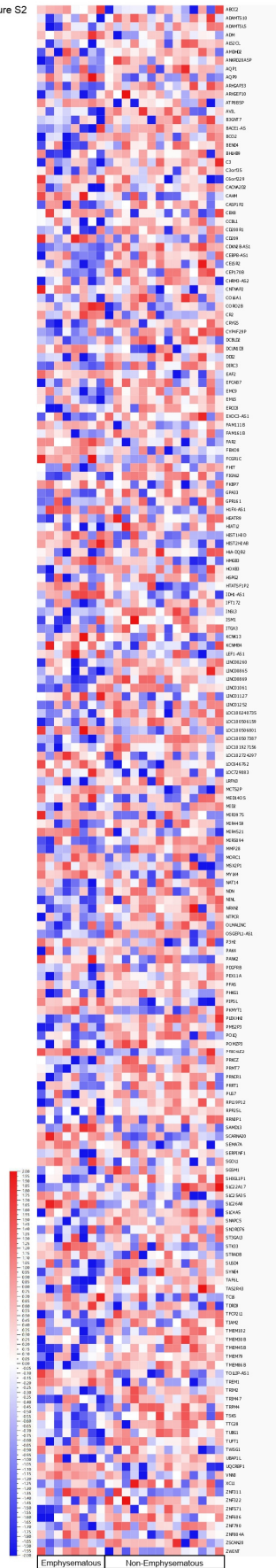

Figure S2: The details of Figure 6 are shown.

Supplement: Supplementary file 1 [file ijms-25-00066-s001.zip › supplementary figures.pdf]
